# How Is the Digital Age Shaping Young Minds? A Rapid Systematic Review of Executive Functions in Children and Adolescents with Exposure to ICT

**DOI:** 10.3390/children12050555

**Published:** 2025-04-25

**Authors:** Reinaldo Maeneja, Joana Rato, Inês Saraiva Ferreira

**Affiliations:** 1 Faculdade de Ciências de Saúde e Desporto, Universidade Save, Maxixe 1301, Mozambique; rmaeneja@unisave.ac.mz; 2 Center for Interdisciplinary Research in Health, Faculty of Health Science and Nursing, Universidade Católica Portuguesa, 1649-023 Lisbon, Portugal; joana.rato@ucp.pt; 3 Health Sciences Faculty, Universidade Europeia, 1500-210 Lisbon, Portugal; 4 Center for Research in Neuropsychology and Cognitive and Behavioural Intervention (CINEICC), Psychological Assessment and Psychometrics Laboratory (PsyAssessmentLab), Universidade de Coimbra, 3000-115 Coimbra, Portugal

**Keywords:** executive functions, Information and Communication Technologies, children and adolescents

## Abstract

**Objectives**: This review assesses how daily exposure to Information and Communication Technologies (ICTs) affects executive functions in children and adolescents and explores the roles of parents in mitigating potential negative impacts on cognitive development and emotional regulation. **Methods**: Following PRISMA guidelines, a systematic search was conducted from 2022 to 2024 using PubMed, Scopus, and Cochrane Library. The study criteria included cohort studies, randomized controlled trials (RCTs), quasi-RCTs, and systematic reviews. Data extraction and risk-of-bias assessments were performed using ROBIS and ROBINS-E tools. Due to the heterogeneity of the results, a narrative synthesis was carried out. **Results**: Ten studies were included for analysis, comprising a total of 231,117 children from nine countries on three continents. Most studies indicated that excessive ICT exposure negatively affects executive functions, particularly working memory, inhibitory control, cognitive flexibility, and attention. Adverse effects were linked to increased screen time, poor sleep quality, and multitasking. However, two studies found no significant association, highlighting the influence of contextual factors like socioeconomic status, parental mediation, and screen content type. Shared ICT use with parents or siblings appeared to reduce negative effects. **Conclusions**: Excessive ICT exposure is associated with impaired executive function development in children and adolescents. Parental supervision and structured ICT use may mitigate risks. Future research should investigate moderating factors, such as socioeconomic status and ICT content, to develop guidelines for healthy digital engagement in youth.

## 1. Introduction

Modern society is shaped by the intensive use of Information and Communication Technologies (ICTs), which have become embedded in everyday life [1,2]. ICT undoubtedly enhances efficiency, productivity, and communication while also saving time [3]. As access to technology and the Internet becomes increasingly universal in many countries, children have begun to use digital technologies at an earlier age [4]. This shift has raised worldwide concerns about the potential impact of ICT exposure on cognitive development, particularly on executive functions (EFs) [5].

Although the theoretical construct discussion continues [6], executive functions are a set of higher-order cognitive processes that include working memory, mental flexibility, inhibitory control, sustained attention, planning, reasoning, and problem-solving. These functions are critical for self-regulation, academic performance, and social adaptation. Childhood and adolescence represent key developmental periods characterized by rapid neuroplastic changes, especially in the prefrontal cortex, where executive function pathways mature. During the first three years of life, a child’s brain can create more than 1 million new neural connections per second, underscoring the importance of early cognitive development [7]. Given the omnipresence of ICT in children’s lives, understanding its impact on EFs has gained growing significance.

The focus on executive functions is due to their critical role in developing self-regulation, learning, and social adaptation during childhood and adolescence [8]. These cognitive skills, which are highly sensitive to environmental influences, have been identified as particularly vulnerable to excessive exposure to Information and Communication Technologies (ICTs) [7,9,10]. Therefore, understanding how ICTs affect executive functions is essential to inform educational practices and strategies for promoting cognitive and emotional well-being [9,11]. In this context, the role of the family becomes particularly relevant, especially concerning parental mediation, in which parents supervise, engage with, and guide their children’s use of ICTs [4,12]. Recent evidence suggests that active parental mediation can mitigate the negative effects of digital exposure by fostering safer, more educational, and developmentally appropriate interactions [12,13,14]. For this reason, the present review chose to examine not only the relationship between ICTs and executive functions, but also the moderating role of the family in this process. Despite the growing number of studies on the impact of exposure to Information and Communication Technologies (ICTs) on the development of executive functions (EFs) in children and adolescents, the literature remains fragmented. Existing studies differ significantly in their methodological designs, age groups analysed, sociocultural contexts, types of ICT considered (educational versus recreational), and the instruments used to assess EFs. This heterogeneity hinders the formulation of robust and applicable conclusions. Therefore, a systematic review is needed to critically synthesise the current evidence, identify patterns and divergences, and contribute to guiding educational practices, parental mediation strategies, and evidence-informed public policies.

The aim of this rapid systematic review is to analyse how the widespread use of ICTs influences the development trajectory of executive functions in children and adolescents, focusing on both neuropsychological and behavioural consequences. Additionally, this review explores the role of families in mitigating potential negative effects through supervision and structured ICT use. Thus, our research question is as follows: How does daily exposure to different types of digital content (educational and recreational) affect the development of executive functions in children and in what ways can parental influence this relationship? By synthesizing recent findings, this study seeks to provide a comprehensive perspective on the complex relationship between ICT exposure and cognitive development during childhood and adolescence. The time window is limited to 2022–2024 due to the rapid advancement of digital technologies and behavioural changes following the COVID-19 pandemic, which justifies the need for more up-to-date and contextually relevant evidence.

### 1.1. Executive Functions and Brain Plasticity

EF development is associated with prefrontal and cingulate cortices changes, alongside neural network reorganization [10,15].

Brain plasticity, the ability to modify neural connections, is most prominent in early life, during critical periods when experience refines neural networks [16,17]. These periods shape behaviour by adjusting excitation–inhibition balances, guided by inhibitory function maturation [18]. Negative experiences can hinder EF development, impacting self-regulation [19]. Reflection and hierarchical neural coordination are necessary for EF development [20,21], allowing children to assess situations, manage conflicts, and develop metacognition [9]. EFs mature progressively, with inhibitory control, cognitive flexibility, and working memory developing through adolescence [22,23]. These functions are crucial for adapting to environmental demands, achieving long-term success, and avoiding risky behaviours typical of youth [24].

### 1.2. Information and Communication Technology (ICT)

The Industrial Revolution propelled Information and Communication Technology (ICT) advancements, shaping modern information systems [3]. The debate around ICT exposure often hinges on the type of digital content, with enthusiasm typically linked to educational use and concern arising from predominantly recreational use. ICTs facilitate access to information and communication within the educational sphere. In preschool education, they have influenced curricula by introducing technology-based interaction through the use of computers in the classroom, serving as a support resource for teachers [25]. In the classroom context, such technology allows educators to focus on one group of children while another remains engaged in playful, meaningful, and learning-oriented activities [25,26], particularly when educational programs are well structured [27].

However, concerns have also been raised regarding the excessive use of ICT [28]. When exposure is mainly focused on entertainment, the effects can be harmful. For example, television has been negatively correlated with parental engagement and children’s language and literacy skills [29]. In the case of video games, the negative impacts tend to be linked to overuse: excessively violent or addictive games have been associated with reduced inhibitory control and increased impulsivity [27]. While ICT supports cognitive, educational, and social development, excessive exposure can lead to negative outcomes such as cyberbullying, sleep deprivation, extended distraction, sexual harassment, obesity, and substance abuse [30,31]. Sedentary lifestyles linked to ICT use increase health risks, including cardiovascular disease [32,33]. A study conducted in selected schools across 11 European countries, involving 11,931 adolescents with an average age of 14.89 years, found that excessive Internet use was associated with unhealthy sleep and eating habits, sedentary behaviour, smoking, and multiple risk behaviours. These associations were observed in 89% of the participants [34].

Given ICT’s ubiquity, concerns about early exposure are rising [4]. The American Academy of Pediatrics (AAP) recommends limiting screen time based on neurodevelopmental stages [15]. ICT exposure during childhood can negatively impact neurodevelopment, executive functions, and brain plasticity [9,35].

### 1.3. Reward Systems and Dopamine: Their Effects in ICTs

The raphe nuclei (RN) establish significant connections with brain areas involved in reward processing, such as the nucleus accumbens (NAc) [36]. The main link between the serotonergic and reward systems occurs through its connection with the dopaminergic system, whose primary neurotransmitter is dopamine (DA) [36,37].

During exposure to ICTs, increased excitability of neurons in the NAc is observed, leading to a more significant release of dopamine in this region [38,39]. With repeated stimulation, the NAc begins to exhibit abnormal activity [38,40], resulting in the continuous activation of the brain’s reward system. Consequently, as individuals use the Internet excessively and for prolonged periods, the reward system becomes increasingly sensitive to digital stimuli [38]. Therefore, exploring the age-specific effects may offer valuable insights into how these relationships evolve throughout childhood.

### 1.4. ICT’s Impact on Planning, Working Memory, and Inhibitory Control

As an EF component, planning involves setting goals and executing strategies [41]. Successful planning requires inhibitory control and working memory [22]. However, ICT’s instant gratification fosters impulsivity, undermining planning and organization [42,43]. ICT distractions affect concentration and attention, key aspects of planning and memory retention [35].

Working memory is essential for language comprehension, learning, and reasoning [44]. It evolves from childhood through adolescence, influenced by attention regulation [19]. ICT overuse can impair cognitive and emotional development, leading to sleep disturbances that affect memory consolidation [45,46]. Exposure to electromagnetic fields from mobile devices may further compromise memory [47,48].

Multitasking, a common ICT practice, weakens memory retention and information processing by overloading cognitive resources [49,50]. Excessive ICT use has been associated with “Digital Dementia”, characterized by cognitive decline and memory loss [51].

Inhibitory control, crucial for self-regulation, develops during childhood and continues to improve into later youth [52]. It plays a role in academic success, health, and behavioural outcomes [53]. Internet addiction, associated with excessive ICT use, reduces grey matter density in the frontal cortex, impairing decision-making and impulse control [38]. This can lead to cognitive impairment, affecting memory and judgment performance [51].

### 1.5. The Role of Family and School in ICT Mediation

Environmental factors influence EFs, requiring parental and educational guidance to ensure healthy ICT use [10]. Home environments offer more autonomy in ICT engagement, while schools provide structured supervision [4]. Raising parental awareness can mitigate ICT overuse and the associated risks [12]. Effective mediation strategies include setting usage limits, monitoring activities and content, and fostering guided interaction [54]. Given that children and adolescents are highly connected to digital platforms [55], it is essential to balance ICT benefits with protective measures to safeguard neurodevelopment [1,35]. However, previous research is still unclear if active parental mediation (e.g., co-viewing and discussing content) might buffer the potential negative effects of recreational digital content and enhance the benefits of educational content.

## 2. Materials and Methods

This systematic review followed Preferred Reporting Items for Systematic Reviews and Meta-Analyses (PRISMA) guidelines. Based on our research issue about how the use of Information and Communication Technologies (ICTs) affects the performance of executive functioning in children and adolescents, an electronic search was carried out on 6 December 2024 using three databases, PubMed, Scopus, and the Cochrane Library, from 2022 to 2024.

The selected time window (2022–2024) was based on the Rapid Systematic Review model, which aims to synthesise recent, relevant, and context-specific evidence. This time frame also seeks to capture post-pandemic effects, marked by changes in ICT use among children and adolescents [56,57].

We used basic Boolean operators and the following search terms: (‘children’ OR ‘adolescents’) AND (‘digital technologies’ OR ‘ICT’ OR ‘screen time’ OR ‘media exposure’) AND (‘executive functions’ OR ‘cognitive development’ OR ‘behavioural outcomes’). Review articles were also searched, and forward and backward searches of the identified studies were carried out.

The selection of studies in this review followed the criteria defined by the PICO model. Of the 202 records identified, only 37 advanced to full-text analysis. The exclusion of the remaining studies was based on the following aspects:

Population (P): Studies that did not involve children and adolescents as the target population were excluded.

Intervention (I): Studies that did not address exposure to ICTs or presented unclear or non-comparable descriptions of the intervention were eliminated.

Comparator (C): Studies were considered ineligible if they lacked a comparison group involving no or reduced ICT exposure.

Outcomes (O): Studies that did not specifically evaluate the impact on executive functions, particularly effects on their development, including neuropsychological and behavioural performances, were excluded.

Additionally, studies employing non-eligible methodologies that were not cohort studies, randomized controlled trials (RCTs), quasi-RCTs, or systematic reviews were excluded, as well as publications written in languages other than English and research protocols without empirical data. Although this rigorous process significantly reduced the final number of included studies (n = 10), it ensured the methodological integrity and robustness of the narrative synthesis.

Only cohort studies, randomised controlled trials (RCTs), quasi-RCTs, and systematic reviews were considered eligible for inclusion, as these study designs are associated with higher methodological quality, greater internal validity, and reduced risk of bias [58,59].

### 2.1. Screening

Two review authors (R.M. and I.S.F.) independently screened the titles and abstracts. Disagreements were resolved through discussion, with a third author (J.R.) consulted to reach a consensus. R.M. then screened the remaining abstracts, while I.S.F. reviewed all the excluded records and resolved any discrepancies, again with the participation of J.R. The same procedure was followed during the full-text screening phase.

**Figure 1:**
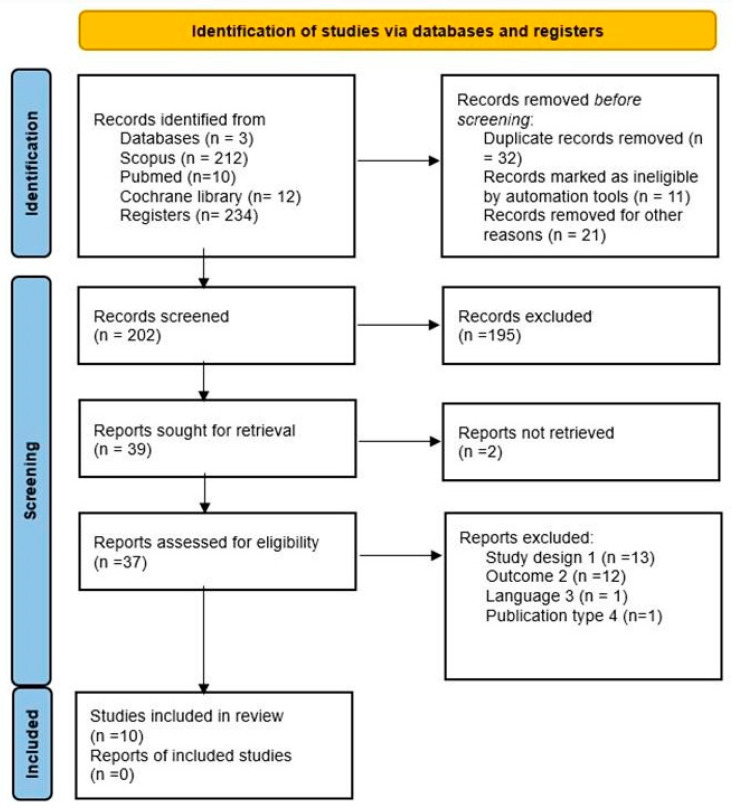
Flowchart of search strategy and selection of reports.

This entire process was carried out using the Rayyan platform, a software program for conducting systematic reviews [60]. After reviewing the complete text, we recorded the reasons for excluding all the excluded studies and documented them in Figure 1.

### 2.2. Data Extraction and Management

The data were extracted (R.M. and I.S.F.), taking into account the following data:

- Report characteristics (including year, authors, and title);
- Study design (including methods, location, groups, and number of participants);
- Characteristics of the participants (including age range 0–18 years and gender);
- Characteristics of the intervention (including context, type of information, and communication technology);
- Comparator characteristics;
- Outcomes and measures evaluated;
- Relevant information to assess the risk of bias.

### 2.3. Assessment of Risk of Bias in Included Studies

**Figure 2:**
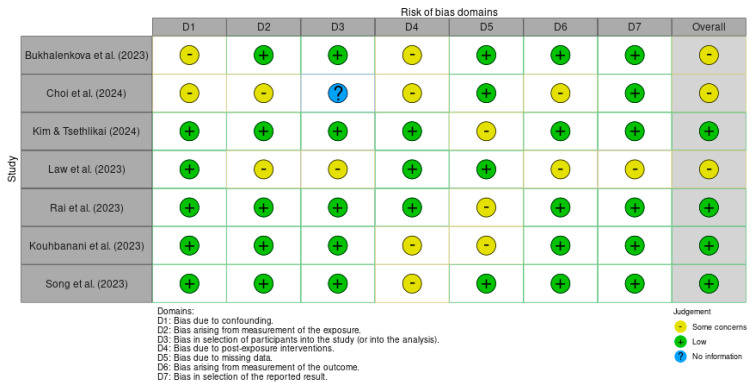
Risk of bias (ROBINS-E) [70,71,72,73,74,75,76].

The risk of bias in the studies included in this rapid review was assessed by R.M. and I.S.F. independently using two tools, ROBIS for systematic reviews [61], as shown in Table 1, and ROBINS-E for cohort studies and experimental studies [62], as shown in Figure 2. A third author (J.R.) was called in whenever necessary to restore consensus.

The ROBIS (Risk of Bias in Systematic Review) tool includes three phases: (1) assessing relevance (optional), (2) identifying concerns with the review process, and (3) assessing the risk of bias [63]. ROBINS aims to assess the risk of bias of an exposure result from cohort studies, case-control studies, and non-randomized studies [62,64,65]. It has seven domains, each addressed through a series of signalling questions that aim to collect relevant information about the study [62]. Due to the heterogeneity of the results, a narrative synthesis was conducted as it allows for a comprehensive integration of findings.

**Table 1:** Tabular presentation for ROBIS results.

Review	Phase 2				Phase 3
	1. Study Eligibility Criteria	2. Identification and Selection of Studies	3. Data Collection and Study Appraisal	4. Synthesis and Findings	Risk of Bias in the Review
Bustamante et al. (2023) [66]	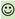	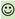	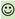	?	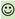
Mallawaarachchi et al. (2024) [67]	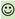	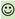	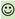	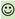	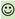
Massaroni et al. (2024) [68]	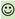	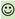	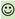	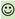	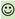

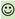
 = low risk; ? = unclear risk.

### 2.4. Data Synthesis

Given the considerable heterogeneity among the included studies, particularly in participant age ranges, the instruments used to assess executive functions, and the types of digital technologies analysed, a narrative synthesis was conducted. This approach is commonly recommended in both rapid and traditional systematic reviews when methodological diversity limits the feasibility of statistical aggregation [56,69].

## 3. Results

Across the three databases used, a total of 234 records were collected: 212 in Scopus, 10 in PubMed, and 12 in the Cochrane Library. After removing duplicates, we screened 202 records, reviewed 37 full-text documents thoroughly, and included 10 studies. Subsequently, we searched for documents that cited any of the initially included studies and the references of the included studies. However, no additional articles meeting the inclusion criteria were found in these manual searches, as shown in the flowchart in Figure 1.

### 3.1. Results of Syntheses

All the included studies address concerns about exposure to Information and Communication Technologies, specifically exposure to televisions, tablets, smartphones, computers, and electronic games, among other Internet-based devices. In total, 10 studies were included in the review, as documented in Figure 1, and the main results of the included studies are summarized in Table 2.

### 3.2. Study Characteristics

The studies included in this review present data collected in nine countries on three continents, as shown in Table 2. We found three studies in Asia, four in Europe, and three in America.

We included five longitudinal cohort studies, two quasi-experimental studies, and three systematic reviews. The studies included in this review as a whole covered a total sample of 231,117 participants, with the youngest aged between 0 and 6 years and the oldest aged between 8 and 12 years.

### 3.3. Risk of Bias in Studies

We found that of the 10 studies analysed, seven had a low risk of bias and seven had some problems, as illustrated in Table 1 and Figure 1.

### 3.4. Exposure to ICT and Executive Functions

This review provides a rapid overview of several neuropsychological instruments used to assess executive functions, with about 10 different measures used between studies to assess subdomains such as working memory (most studied), inhibition control, cognitive flexibility, planning, and organization (Table 3). Our review also found that eight of the then studies included television as one of the studied devices. Among the ten studies analysed, one suggests that reducing exposure to ICTs improves sleep quality and, consequently, cognitive functioning [71]. Another study associates ICT exposure with cognitive impoverishment [67,68,73,74], especially linking excessive use of TVs, smartphones, computers, and tablets to impaired cognitive flexibility [70]. Moreover, limiting ICT exposure—particularly time spent on video entertainment—has been shown to improve inhibitory response control [76]. Environmental conditions, especially exposure to ICTs, have also been reported to affect executive functions negatively [75]. However, two studies found no significant association between ICT exposure and executive function performance [66,72].

Regarding child neurodevelopment by age group and the type of ICT content—whether educational or entertainment-oriented—four of the ten studies analysed focused on infants, preschoolers, and early school-age children, aged between 0 and 6 years [66,67,68,72]. Among these, three studies reported exposure to ICT in the context of entertainment [66,67,68], while one study assessed exposure to educational content [72].

One study examined preschool children aged 3, where exposure was linked to both educational and entertainment content [74]. Another study focused on children in preschool and early school age, between 5 and 7 years old, with ICT use also occurring in an entertainment context [70].

Two studies analysed children in middle childhood, aged 9 years, identifying exposure to ICT in entertainment [71,73]. One study investigated children in middle to late childhood, aged 9 to 10 years, with exposure also related to entertainment [76]. Finally, one study assessed children ranging from early school age to middle/late childhood, aged 8 to 12 years, with exposure again situated within an entertainment context [75].

### 3.5. Family Interactions and Children’s Use of Information and Communication Technologies

Seven of the ten studies included in this review analyse family interactions in ICT consumption and their implications for child development and family dynamics. Children who watched video content and played video games with their siblings insignificantly improved inhibitory control over the year compared to those who did so alone [70]. Similarly, co-using ICTs with others was associated with better cognitive outcomes [67]. Moreover, positive parenting practices were associated with less screen time use in children [71,72]. Kim and Tsethlikai (2024), Law et al. (2023), and Massaroni et al. (2024), in turn, primarily focus on exposure to ICT duration, with limited discussion on its potential influence on the socioeconomic conditions of the children’s families [72,73]. Additionally, parents’ reports indicate that preschool children (approximately 5 years old) with more than one hour of daily screen time demonstrated increased vulnerability in cognitive domains [68,74]. Although only three studies explore the implications of ICT use for child development, they do not address family interactions [66,75,76].

## 4. Discussion

This review aimed to analyse how the omnipresence of Information and Communication Technologies (ICTs) influences the development of executive functions in children and adolescents, highlighting the potential neuropsychological and behavioural effects of exposure. Furthermore, we explored how families can mitigate these effects by supervising and regulating ICT use.

### 4.1. Impact of ICT Exposure on Executive Functions

Technological advancements in recent decades have led to a greater engagement of children and adolescents with screen-based ICTs, while simultaneously decreasing their contact with nature. Most available evidence on this reality comes from high-income countries [77], a trend also corroborated by our Rapid Systematic Review, since the included studies report data from countries such as Australia, Canada, South Korea, Spain, Italy, Iran, Russia, and Singapore, highlighting a shift away from natural to digital environments [78].

Of the ten studies included, eight report the negative impact of ICTs on executive functions, especially working memory [67,70,73,74,76,79], inhibitory control [67,71,73,74], cognitive flexibility [67,71], attention [67,73], and global cognitive functions [68]. These findings align with previous research indicating that ICT use can overload memory [80] by exceeding an individual’s attentional resources, leading to decreased memory retention and increased stress levels [51]. Since working memory plays a vital role in learning, enabling the selection, comparison, and organization of relevant information before it is transferred to long-term memory [81], such an overload can have significant educational implications. Previous research suggests that the ubiquity of ICTs affects memory processes [82], as individuals who rely on digital devices for information storage are less likely to retain it long-term, potentially leading to memory loss [51]. In addition, according to the studies here screened, an excessive reliance on technology, even for simple tasks, can impair memory retrieval [51]. Since memory is an essential cognitive function [83], its proper functioning is the basis for effective learning and social integration [84].

Regarding the impacts of ICTs on inhibitory control—one of the subdomains here identified as being affected—their pervasive presence influences brain structure and function, significantly affecting both attention and self-regulation [42]. Excessive ICT use has been linked to weakened inhibitory control, increased impulsivity, and impaired self-regulation, as observed in previous studies [85]. Recent research [38] also reinforces these findings, highlighting a reduced ability to resist distractions. These effects arise as the brain becomes conditioned to immediate rewards [42]. Impulsivity is often characterized by acting in the heat of the moment, struggling to maintain attention on tasks, and failing to plan or think carefully [86]. This occurs because an overactive, impulsive system triggers quickly, which interferes with the reflective thinking essential for cognitive control [87]. In our review, only two studies emerged within the adolescence period, with children up to 12 years old [75,76]; however, given the latest World Health Organization report, internet use among adolescents increased from 7% in 2018 to 11% in 2022, in which more than one in ten adolescents showed signs of problematic social media behaviour [88].

Additionally, a recent cross-sectional study indicates that adolescents with low self-control are more likely to respond immediately to the notifications they receive because they want immediate gratification and are often unable to recognize negative consequences [89]. These seem to be sufficient reasons to continue studying adolescents’ ICT behaviours. Excessive exposure to ICT has been linked to increased anxiety levels [90], which can result in emotional distress [91]. This, in turn, may lead to problems with concentration, attention, and memory, as well as challenges in managing impulses, planning, and organization [5,92]. For example, the excessive use of ICTs, especially in multitasking demands, is associated with reduced sustained attention [93,94], which impairs the development of the prefrontal cortex [95].

Considering the two studies that report no association between exposure to ICTs and the functioning of EFs [66,72], they state that if we want to determine the effect of the overall use of screen time on children’s EFs, it is necessary to consider other factors related to context and development [66]. In addition, the results of two studies were presented; one shows a positive association between exposure to ICTs and EFs [82], and another [83] shows that regular exposure to screens at 4 months predicts worse inhibition performance at 14 months. However, despite this evidence, other studies [61,84] indicate no significant relationship between the use of various screen-based media devices and EF measures. One justification is based on the fact that studies analyse the relationship between EF and the use of screen time across different development stages [66]. This could be an acceptable approach, since executive functions develop progressively rather than uniformly throughout childhood and adolescence [44,96,97]. Different periods of development have different sensitivities to environmental influences, such as exposure to technology [24,27]. Thus, studying the impact of screen time at different ages can lead to varied results since children may respond differently to the same conditions depending on their stage of maturation or even due to pre-existing traits such as temperament, which can influence the degree to which children engage with ICTs [98]. With regard to the other study conducted, [72] states that screen time duration is not significantly related to later difficulties in socioeconomic status, and the justification found for these results lies in the sample not adequately representing children from low socioeconomic families. We believe this reasoning is justified, insofar as the representativeness of the sample is a critical factor in any research, given its potential to impact the results, as demonstrated in the study conducted [72]. However, although we find the justification reasonable, we can question the validity of the reasoning, given that there are studies that show that low socioeconomic status per se has a negative impact on the development of EFs and other cognitive skills throughout childhood and adolescence, because it compromises the structural and functional maturation of the brain and neuroendocrine profiles due to exposure to factors such as chronic stress [99].

Nonetheless, within the scope of our discussion, particularly the issue of age group differentiation versus whether ICT exposure involves educational or entertainment content, the findings suggest that exposure to Information and Communication Technologies (ICTs) has a differentiated influence on the development of executive functions (EFs) depending on the child’s age and the type of content consumed. This differentiation is consistent with the trajectory of child neurodevelopment, according to which there are periods of heightened brain sensitivity that render certain developmental stages more or less vulnerable to environmental effects, such as digital technology use [96,100,101,102,103].

Between 0 and 2 years of age, a stage characterised by intense synaptic formation and accelerated development of the prefrontal cortex, the most relevant cognitive milestones are attention and the onset of memory [104]. At this stage, exposure to digital content, especially without human interaction, may compromise the quality of stimuli required for the consolidation of these abilities. The studies by Bustamante et al. [66] and Massaroni et al. [68], which involved this age group, reported either negative impacts of screen time on cognitive and language development or the absence of statistically significant associations with EFs, suggesting that the type and context of use may either mitigate or exacerbate the effects.

From 3 to 5 years of age, when inhibitory control and cognitive flexibility begin to emerge, educational content appears to have more positive effects on EFs [22]. This was observed by Kim and Tsethlikai [72], whose results indicated that higher levels of exposure to educational content predicted fewer difficulties in EFs. This contrasts with findings by Bukhalenkova et al. [70], who reported a decline in cognitive flexibility among children who watched videos accompanied by their parents—suggesting that co-viewing, when not intentionally mediated, may not yield the expected benefits.

In the 6–8-year age range, a critical period for the consolidation of planning, organisation [105], and cognitive monitoring, studies by Law et al. [73] and Rai et al. [74] point to the risk of cognitive impoverishment resulting from the replacement of real-world interactions with digital ones and to the negative impact of excessive screen use on specific domains of cognitive development. Among children aged 9 to 12 years, a phase marked by significant advances in self-regulation and metacognition, the evidence presented by Song et al. [76] and Kouhbanani et al. [75] suggests that prolonged exposure to entertainment-based content may alter brain functional patterns and compromise daily executive performance. These findings underscore the need for public health strategies aimed at reducing excessive exposure to recreational screen media.

Furthermore, data from Choi et al. [71], which link shorter sleep duration to longer screen time and reduced variability in usage patterns, indicate that the effects of ICT exposure are not only direct but also occur through changes in behaviour and lifestyle, which in turn have indirect consequences on executive functioning. In summary, age-based analysis reinforces the importance of adopting developmentally sensitive approaches, considering that ICTs can both stimulate and inhibit cognitive skills, depending on the content, duration of exposure, context, and maturational stage of the child.

### 4.2. Family Interactions and the Use of ICT by Children and Adolescents

Seven studies analyse family interactions in the consumption of ICTs and their implications for child development and family dynamics. For example, [70] presented results that point to the benefits of shared ICT use, as it helps develop inhibitory control [58] and improves working memory, inhibition, and shifting [67]. Shared use plays a mediating role, helping to develop resilience to the risks of harm resulting from large exposure [106]. Choi et al. discuss the issue of positive parenting in their results as being associated with lower screen time use in children [71]. Active parental mediation is recognized as an essential influence in reducing adolescent risk behaviours [13]. The recent literature reports that the best way to use ICT in childhood and adolescence is through supervision, as this can also help protect children from addiction through close parental monitoring and support [107].

Children whose parents set clear rules and consistently monitor Internet use tend to have a lower risk of ICT addiction [5]. On the other hand, the children of parents who do not control Internet use have a high risk of screen addiction [5,108]. For this reason, a lack of rules for ICT use is a main risk factor [109].

Kim and Tsethlikai [72], Law et al. [73], and Massaroni et al. [68], despite not directly addressing family dynamics in the context of ICTs, discuss the influence of the sample on the results. They state that the lack of significance between ICT exposure and EFs in one study was partly due to the fact that the sample did not adequately represent children from families of low socioeconomic status [72]. The findings that family income mediates the association between children’s screen time and executive functioning [73] and that parental reports suggest that preschool children with screen time exceeding 1 h showed greater vulnerability in cognitive areas [68] highlight the impact of screen time on cognitive development. As we mentioned earlier, the social conditions of families can interfere both positively and negatively, as socioeconomic status is considered a risk factor for problematic behaviour related to the Internet, since parents with a high socioeconomic status could give their children more guidance and clear instructions for regulated use [13]. Spending too much time using digital media takes time away from real-life exploration and play activities that are decisive for early childhood development, and parental mediation is an important factor in protecting children from the risks they face when using ICTs [110].

Rai et al. [57] discuss the dynamics of family relationships and ICTs, but unlike the other studies included in this review, the results show that the total duration of screen time, video viewing, and co-use were negatively associated with some cognitive domains, which suggests that it is not just the amount of screen time that matters, but also the type of content and the context in which it is used. Studies highlight the importance of considering this detail when discussing ICTs and executive functions [111,112]. Educational content, for example, has positive associations with aspects of cognitive development [111], whereas entertainment content, such as videos, programs, films, has been linked to negative associations with cognitive development measures [112].

As noted previously, in the context of the family, shared activities are more likely to create a sense of “we” that supports family cohesion [14], providing emotional support and behavioural models that promote autonomy and self-control [113], since the cohesion and adaptability of the family has a protective role [114].

Previous research, such as that of Schilder et al. [115], which investigated the effectiveness of a school-based intervention on online risk awareness and behaviour, reinforces the idea that raising awareness is a necessary step toward behavioural change. This evidence underscores the importance of adult-mediated interventions, particularly those delivered in school settings, and supports our review’s findings on the critical role of active mediation and co-viewing in moderating early adolescents’ exposure to digital content.

One important aspect to consider when analysing this review is that some studies, such as those by Kim and Tsethlikai [72], Law et al. [73], and Rai et al. [74], relied predominantly on parental self-reports to assess both children’s exposure to ICT and indicators of executive function performance. This type of measurement is prone to several biases, including recall bias, social desirability, and subjective interpretation, which may compromise the reliability of the data collected. In this regard, Sina et al. [116] found no significant associations between ICT exposure and executive functions based on self-reported data, in contrast with earlier studies that used standardised assessment scales.

Another noteworthy point is that the evidence provided by Bustamante et al. [66], Mallawaarachchi et al. [67], and Massaroni et al. [68] is based mainly on cross-sectional studies, which limits the ability to infer causal relationships between ICT exposure and the development of executive functions. Even studies that included biological mediators, such as the one by Kouhbanani et al. [75], maintained a cross-sectional design, thus restricting the temporal interpretation of the observed effects.

This predominance of self-report measures and non-longitudinal designs is particularly relevant given the complexity of executive functions, whose assessment requires multi-method approaches sensitive to developmental variations throughout childhood. Therefore, it is recommended that future research adopt mixed-method designs, combining objective behavioural measures, observational records, and longitudinal assessments to capture better the developmental trajectory and the determinants of executive functioning in digital contexts.

### 4.3. Policy Implications and Practical Interventions

Given the documented impact of excessive ICT exposure on the development of executive functions in children and adolescents, the findings of this review carry clear policy implications that decision-makers in the health, education, and communication sectors should consider. Firstly, developing age-based national guidelines for healthy screen use is recommended, including daily exposure limits and the promotion of educational digital content. These guidelines should be disseminated through public awareness campaigns, focusing on the role of active parental mediation and intentional co-use of digital devices.

Furthermore, the data reinforce the need to incorporate digital literacy into school curricula, using approaches that foster self-regulation, sustained attention, and responsible technology use from early education. It is also proposed that teachers and health professionals be trained to recognise risk signs associated with excessive ICT use and to guide families based on evidence-based practices. Finally, it is imperative to consider the influence of socioeconomic status on ICT exposure and parental mediation, which justifies the implementation of targeted digital inclusion policies and psychosocial support in vulnerable contexts. These combined measures are essential to mitigate the risks identified in this review and to promote healthier and more adaptive use of technology during childhood and adolescence.

### 4.4. General Limitations

The evidence included in this rapid systematic review has some limitations that should be considered when interpreting the results. Firstly, most of the studies were conducted in high-income countries, limiting the generalizability of the results to low-income populations or disadvantaged socioeconomic contexts. In addition, there was heterogeneity in the neuropsychological instruments used to assess executive functions, as illustrated in Table 3, which affected the comparability of the results between the studies. Another noteworthy aspect was that two of the included studies found no significant association between exposure to ICTs and executive function performance, suggesting that contextual factors, such as parental mediation and the type of ICT content, may modulate the effects observed. It is important to acknowledge that several studies included in this review relied on parent or caregiver self-reports as the primary source of data on executive function performance, which may introduce biases and limit the accuracy of the results. Moreover, the predominance of cross-sectional study designs compromises the ability to establish causal relationships between ICT exposure and cognitive outcomes. Another relevant limitation concerns the complexity and breadth of the concept of executive functions. The included studies assessed various domains—such as inhibitory control, cognitive flexibility, planning, and working memory—using distinct measurement tools as illustrated in Table 3. This variability limits the comparability of findings and complicates the generalization of conclusions. These methodological differences should be considered when interpreting the overall strength and applicability of the evidence.

These limitations highlight the need for caution when generalizing the results to different populations and contexts.

### 4.5. Limitations Associated with the Processes Used in the Review

This systematic review has several limitations which should be considered when interpreting the results. Firstly, although we followed the PRISMA 2020 guidelines and carried out a comprehensive search in three databases (PubMed, Scopus, and Cochrane Library), it is possible that relevant studies were omitted. This may be due to the exclusion of studies published in languages other than English or in journals not indexed in the databases consulted. In addition, this review was limited to studies published in the last two years (2022–2024), which may have excluded important evidence from previous studies, especially in a rapidly evolving field of research such as the impact of ICT on cognitive development.

An additional limitation concerns the lack of prior protocol registration in dedicated platforms such as PROSPERO. While we acknowledge that prior registration is a recommended practice to enhance transparency and reduce bias, this step was not undertaken due to the time-sensitive nature of this rapid review. Nonetheless, we recognize this as a methodological limitation, as the absence of a pre-registered protocol may affect the reproducibility and perceived rigor of the review process.

Additionally, the inability to perform a meta-analysis is due to the high heterogeneity among the included studies observed in study designs, participant age ranges, assessment instruments used, and the types of exposure to digital technologies. Nevertheless, narrative synthesis is a valid and appropriate approach for integrating findings in contexts of substantial methodological variation [56,117].

### 4.6. Implications of the Results for Future Research

The results of this systematic review have significant implications for future research, as our findings suggest that excessive exposure to ICT can impair the development of executive functions in children and adolescents, particularly in domains such as working memory, inhibitory control, and cognitive flexibility. It is therefore essential that parents, educators, and health professionals promote a balanced and supervised use of ICT. Strategies such as setting screen time limits, selecting educational content, and active parental mediation can help mitigate the negative effects. In addition, activities that involve shared use of ICTs (for example, watching videos or playing games with parents or siblings) can be beneficial, as they have been associated with better cognitive outcomes in some studies.

Based on this review, we identified some gaps that need to be addressed in further studies, such as the need for future studies exploring the effects of ICT in low-income populations and in different cultural contexts, since most of the current evidence comes from high-income countries. In addition, future research should investigate the mechanisms underlying the effects of ICT on the development of executive functions, including the role of factors such as the type of content consumed, the duration of exposure, and parental mediation. Longitudinal studies with robust designs and standardized instruments to assess executive functions are essential to provide more consistent and comparable evidence, and we also think there is a need for studies that explore the impact of ICT at different stages of development, from early childhood to adolescence, to understand how the effects vary over time.

## 5. Conclusions

Our data review highlighted the influence of ICTs on executive functions in children and adolescents, revealing that excessive exposure significantly impacts multiple components, such as working memory, inhibitory control, cognitive flexibility, and organization. These effects are strongly supported by studies linking the prolonged use of digital devices to reduced attention, increased stress, and impaired fundamental cognitive skills.

However, it is important to recognize that the impacts vary according to contextual factors, such as children’s stage of development, the type of screen content consumed, and parental mediation. Supervision and the shared use of ICTs emerge as essential practices to mitigate the negative effects, promoting a healthier and more constructive use of these technologies.

Our review also identified gaps in the existing recent research, such as the need for more representative studies of populations with low socioeconomic status and a more detailed analysis of the influence of individual variables such as temperament and family context. Adolescence also deserves to be studied more extensively, given the growth in ICT use at these ages, even for school purposes. Future studies should adopt more comprehensive approaches to bring to the surface the aspects that guide the complexity of this field of knowledge.

Based on the findings of this rapid systematic review, it is possible to provide more specific recommendations tailored to different age groups and types of exposure to digital technologies. The evidence suggests that the effects of ICTs on executive functions are influenced not only by screen time but also by the content, usage context, and the presence or absence of adult mediation. From birth to age 2, it is generally discouraged, except for interactive video calls, as the brain relies heavily on real-life stimuli. Between the ages of 3 and 5, limited educational content may be beneficial if co-viewed with caregivers, while passive content should be avoided. For ages 6 to 8, screens should be used mainly for educational purposes with supervision, without replacing play or social interaction. From 9 to 12, unsupervised use of social media and video games can impact executive functioning, making balanced digital habits and open communication essential. For adolescents 13 and up, increasing autonomy requires ongoing support to foster critical use of technology, self-regulation, and balance across digital and offline activities.

Across all age groups, it is recommended to adopt active parental mediation practices—such as discussing content, watching together, and setting boundaries—and to promote the intentional, pedagogical use of ICTs, particularly in educational settings. These strategies provide practical and contextualized guidance that can support family, school, and community interventions aimed at fostering healthy and balanced ICT use throughout childhood and adolescence.

## Figures and Tables

**Table 2:** Main characteristics of the included studies.

Author (Year)	Study Design	Country	Sample Size	Age, Mean	Gender	Families, Schools, and ICT	Outcomes
Bukhalenkova et al. (2023) [70]	longitudinal cohort study	Russia	490	children over a year (from 5–7 years old).	male/female	In total, 53.7% of children watched cartoons, movies, and videos with siblings, one-third watched alone, and 15.6% with parents.	Children who watched video content with their parents exhibited a decline in cognitive flexibility over the year.
Bustamante et al. (2023) [66]	meta-analysis	Spain	6922	children aged from 0 to 6 years	does not apply	Since few studies have addressed ICT exposure at home and in school, the aim was to estimate the association between screen time and EF.	No statistically significant association in the relation between overall time use and EF or in the selected moderators
Choi et al. (2024) [71]	longitudinal cohort study	Canada	110	average age of children = 9.14 years	unspecified	Positive parenting style was associated with fewer hours spent on screens.	Shorter sleep duration correlated with increased screen time and lower variability in usage over time, impacting EFs.
Kim and Tsethlikai (2024) [72]	longitudinal cohort study	South Korea	2.150	average age of children = 5.59 months (SD = 1.22 months)	male/female	Cross-lagged paths demonstrated that an increase in the extent to which children engaged in educational ST was significantly related to a decrease in EF difficulties.	Screen time duration did not predict EF difficulties one year later. However, higher levels of educational ST predicted fewer EF difficulties.
Law et al. (2023) [73]	longitudinal cohort study	Singapore	437	the mean (SD) age at follow-up was 8.84 (0.07) years	male/female	Screen time represents a measurable contextual characteristic of a family, an indicator of the quality of interaction between parents and children.	Screen use may be a proxy for cognitive impoverishment due to the displacement of social interactions in real life.
Mallawaarachchi et al. (2024) [67]	systematic review and meta-analysis	Australia	176.742	early childhood (birth to <6 years)	does not apply	Co-use with others (e.g., parents and siblings) was associated with better cognitive outcomes	TV exposure was associated with poorer cognitive outcomes. r values ranged from −0.16 to 0.14
Massaroni et al. (2024) [68]	systematic review	Italy	32.274	children between 0 and 7 years	does not apply	Watching television without guidance reduced verbal activity and increased the risk of developing a delay in language acquisition.	Preschool screen time had negative effects on children’s cognitive and language development.
Rai et al. (2023) [74]	quasi-experimental	Canada	44	age of children = 3.5 years (± 0.3)	male/female	Children spent on average 103.5 min/day (SD = 59.2) engaged in screen time, 24.9 min/day (SD = 29.5) using mobile screen devices, and 48.1 min/day (SD = 30.5) co-using with an adult.	Excessive screen time may be detrimental to some domains of cognitive development.
Soltani Kouhbanani et al. (2023) [75]	quasi-experimental	Iran	133	being 8–12 years old	male/female	Screen time of children was equal to 225 min (SD = 72 min.). Home EF environment score was equal to 39.65 (SD = 7.26)	The function of brain waves is affected by environmental factors. Hence, the children’s daily EF was influenced.
Song et al. (2023) [76]	longitudinal cohort study	USA	11,815	aged 9–10 years (subgroup 1 = 119.16 ± 7.47 subgroup 2 = 118.92 ± 7.5, in months)	male/female	The week-average screen time significantly increased by 0.73 h	The findings suggest that public health strategies aimed at decreasing excessive time spent by children on video-entertainment-related SMA are needed.

**Table 3:** Neuropsychological tests used in the studies and the cognitive domains assessed.

Author	Type of Device	Executive Functions or Sub-Domain	Neuropsychological Tests
Bukhalenkova et al. (2023) [70]	TVs, smartphones, computers, and tablets	Working memory Cognitive inhibition	NEPSY-II subtest
		Cognitive flexibility	Dimensional Change Card Sort task
Bustamante et al. (2023) [66]	Screen-based devices like TVs, computers or laptops, smartphones, and tablets.	Working memory	Not applicable (Meta-analysis)
		Cognitive inhibition	
		Cognitive flexibility	
Choi et al. (2024) [71]	TVs, tablets, computers, and phones	Executive function	Learning, Executive and Attention Functioning (LEAF)
Kim and Tsethlikai (2024) [72]	TVs, tablets, smartphones, and computers	Executive function	Executive Function Difficulty Screening Questionnaire
		Plan/organize	Plan/Organize subscale
		Inhibitory control	Behavioral Control subscale
Law et al. (2023) [73]	Mobile electronics	Naming inhibition, shifting, and working memory	NEPSY-II subtest
		Attention and executive functioning	Child Behavior Checklist (CBCL), General Executive Control Problems scale.
Mallawaarachchi et al. (2024) [67]	Television, videos, DVDs, or movies	Working memory, inhibition, and shifting	Not applicable (Systematic Review and Meta-analysis)
Massaroni et al. (2024) [68]	Televisions, computers, and smartphones	Working memory	Not applicable (Systematic Review)
Rai et al. (2023) [74]	Television shows via YouTube and playing electronic games	Working memory	Forward and backward span phases of a word span test
		Inhibitory control	Head toes knees shoulders (HTKS)
Kouhbanani et al. (2023) [75]	TVs, laptops/computers, Smartphones, and tablets	Executive function	EEG
			Barkley Deficits in Executive Functioning Scale (BDEFS)
			Home executive function environment (HEFE)
Song et al. (2023) [76]	Video entertainment	Inhibitory control	Behavioral Inhibition/Activation System (BIS/BAS)
			ABCD modified UPPS-P Impulsive Behavior Scale

## Data Availability

The original contributions presented in the study are included in the article, further inquiries can be directed to the corresponding author.

## Abbreviations

ICT	Information and Communication Technology
EFs	executive functions
